# Supplementary material for: Are carbon emissions trading and green financial instruments synergistic? -Comprehensive quantitative research based on content analysis
Source: PLoS One. 2024 Mar 7;19(3):e0298601. doi: 10.1371/journal.pone.0298601 (PMC10919601; doi:10.1371/journal.pone.0298601)
Supplement: S1 Data — (ZIP) [file pone.0298601.s001.zip › Data/地级市Z_F工作报告.zip!/╦─┤¿/╣π░▓╩╨/└┤╫╘╣1⁄2╓┌║┼í╛┬φ┐╦╩2╛▌═°í┐_2018.docx]

各位代表：

现在，我代表市人民政府，向大会报告工作，请予审议，并请市政协委员提出意见。

2017年为新时代广安更大发展打下了更加坚实基础

2017年，新一届市人民政府以习近平新时代中国特色社会主义思想为指导，在省委、省政府和市委的领导下，在市人大、市政协的监督支持下，深入学习和贯彻落实党的十九大精神和省第十一次党代会精神，把握稳中求进工作总基调，坚持新发展理念，以供给侧结构性改革为主线，扎实推进“三大发展战略”和军民融合发展战略，统筹做好稳增长、促改革、调结构、惠民生、防风险各项工作，逢山开路、遇水架桥、披荆斩棘、勇往直前，保持了连续多年来稳中有进、稳中向好发展势头。全年实现地区生产总值1173.8亿元、增长8.1%，地方一般公共预算收入达到71.2亿元、增长15.9%，圆满完成市五届人大一次会议确定的全年目标任务，推进“两个跨越”、建设美丽繁荣和谐广安取得新的重要进展。

投资消费保持较快增长。扎实推进“项目年”工作，全社会固定资产投资1513.3亿元、增长16.5%，其中民间投资791.4亿元、增长12.6%。集中开工4批次395个重大项目，759个省市县重点项目完成投资1056.6亿元。投资结构得到优化，工业投资达到309.8亿元、增长11.1%，广深产业示范基地一期、比亚迪云轨生产基地等重大项目开工建设，新青阳药业、利尔化学草铵膦等项目加快推进；工业技改投资达到138.1亿元，增长12.9%；122个水利项目纳入国家或省“十三五”规划，新开工水利项目24个，在建中型水库5座；第三产业投资大幅增长，建成一批大型城市商贸综合体。社会消费品零售总额525亿元、增长12%。

产业发展取得新进展。三次产业结构调整为14.5：46.5：39。规模以上工业增加值增长9.3%，9个重点产业实现产值1319.3亿元、增长34.5%。华蓥市电子信息产值达到190.8亿元，岳池县生物医药产值达到63.9亿元，邻水县装备制造产值达到151.3亿元，武胜县农副产品加工产值达到162.9亿元，前锋区轻纺产值达到117.9亿元，广安经开区精细化工产值达到52亿元。新建省级科技企业孵化器和众创空间3家、省市级企业技术中心10家，实施专利转化项目40项。培育规模以上服务业企业46户，服务业增加值457.9亿元、增长10.3%，其中电子商务、现代物流等新兴先导型服务业实现增加值164亿元、增长11%。广安区纳入全省首批服务业强县。全市旅游总收入达到350.7亿元，增长16.1%。金融业持续发展，存款余额新增166.2亿元、增长9.9%，贷款余额新增112.3亿元、增长13.7%，实现直接融资101.7亿元。

农业农村经济稳定发展。农村土地确权登记颁证率达到98.68%，土地经营权流转面积86.93万亩，农村集体资产股份合作制改革试点稳步推进。深化农业供给侧结构性改革取得新进展，新建现代产业基地26.4万亩，发展农产品初加工企业72户、精深加工企业50户，农产品区域公用品牌“华蓥山”成为四川省十大优秀品牌。粮食生产保持稳定增长。

改革开放取得新成效。扎实推进“三去一降一补”，关闭煤矿13户、化解煤炭产能131万吨，压减水泥产量278.2万吨，商品房库存消化周期降至7.2个月，为企业减轻税费14.28亿元。深化“放管服”改革，取消行政审批事项12项，6189项行政权力和6123项责任事项纳入清单管理，政务服务一体化平台上线运行。全面完成国家中小城市综合改革试点建设任务。招商引资取得实效，国内省外到位资金423亿元。广安（深圳）产业园加快建设，渠江·云谷孵化园规划展览馆建成投用，渝广合作战略协议有序实施，新组建天津高新区（广安）创新示范园项目合资公司，中关村（广安）高新产业示范基地建设稳步推进，成功引进温差芯片、无损压缩智能芯片等高端产业项目，与西安航光合作建设国内首个北斗数字化智慧城市。外贸进出口总额达到20亿元，实际利用外资4600万美元，跨境商品体验中心及川东北首个公用型保税仓建成运营，新增生产型出口企业8户。

城乡基础设施持续改善。全市拓展城镇面积8平方公里，常住人口城镇化率提高1.6个百分点。投资37.5亿元推进海绵城市、地下综合管廊等市政基础设施和公共服务设施建设，神龙湖建设、万盛片区雨污分流改造及道路综合整治加快推进，西溪河城区段截污干管及配套工程、官盛湖建设基本完工。开工改造城镇危旧房棚户区16209套，农村危房改造开工10199户、竣工8623户。推进50个特色集镇和14个“百镇建设行动”试点镇建设，建成幸福美丽新村409个。大龙大桥、省道208邻水段、省道406华蓥段、国道212武胜段一期工程等开工建设，过境高速公路东环线及渝广高速支线进场施工，清溪口渠江大桥、官盛渠江大桥等加快建设，广安客运枢纽站、武胜客运中心、邻水客运中心投入运营。新（改）建县乡公路321公里、村道1186公里。

脱贫攻坚圆满完成全年任务。坚持把精准扶贫精准脱贫作为“头等大事”来抓，48232名贫困群众脱贫，266个贫困村退出，广安区高标准通过国家验收，前锋区、华蓥市通过省上验收。整合产业扶贫资金3.9亿元，发放扶贫小额信贷19.2亿元，发放教育救助金4.7亿元，为贫困群众报销（补助）住院医疗费用3.3亿元，为5.82万名低保兜底对象发放低保金1.47亿元，完成易地扶贫搬迁5603户、16762人。“十看五帮三机制”防止返贫经验受到省委肯定，再次创造了“广安经验”。广安市荣获“全省脱贫攻坚先进市”，广安区、前锋区、华蓥市获得“全省脱贫攻坚先进县”荣誉。

人民群众生活日益改善。城乡居民人均可支配收入达到30616元、13655元，分别增长8.5%、9.4%。民生支出达到184.7亿元，占一般公共预算支出69.4%，省定民生工程104项全面完成，市定民生工程22项顺利实施。新增城镇就业4.71万人，失业人员再就业1.26万人，城镇登记失业率3.6%。城乡居民基本医疗保险参保覆盖率达到99%。新建公办幼儿园7所，实施乡镇中心校标准化建设122所，改造农村义务教育薄弱学校162所，广安区、前锋区义务教育均衡发展通过国家认定。广安职业技术学院被列为省优质高职院校培育建设单位。语言文字工作通过省政府督导评估。市人民医院儿科及第二住院大楼完成主体工程，成都中医药大学附属广安医院挂牌成立，建立市县乡医疗联合体11个，计划生育和公共卫生服务取得实效。新增养老床位2600张，新建城乡社区日间照料中心78个。群众性文体活动蓬勃开展，实施“优秀剧目进广安”、演出精品剧目15个，全面启动健康跑、书法、足球“三进校园”活动，成功举办广安国际红色马拉松赛。顺利通过全国文明城市复查。

生态建设和环境整治力度加大。中央和省级环保督察问题整改完成率分别达到92.1%、87.3%。打好污染防治“三大战役”，建成“洁净水”项目155个，创新河长制“1+4+1+N”河湖管护模式，国控、省控断面水质全部达标，城市和农村集中式饮用水水源地水质达标率分别为100%、92.6%；实施减排、抑尘、压煤、治车、控秸行动，城区空气质量达标天数310天、达标率上升5.7个百分点，获得省空气质量改善激励资金1001万元；严格执行生态保护红线制度，土壤环境质量总体稳定。营造林26.5万亩，新增森林11.2万亩。华蓥山区山水林田湖草生态保护修复工程纳入全国第二批试点，获得中央首批基础性奖补资金10亿元。防灾减灾、气象等工作进一步加强。

社会大局保持和谐稳定。“七五”普法深入推进，“法律七进”持续开展，“一村一社区法律顾问”制度有序推行，全社会法治观念增强。信息预警处置、社会稳定风险评估等机制进一步健全和得到落实，矛盾纠纷排查化解工作扎实深入，民间投融资依法治理全面加强，全年未发生造成较大负面影响的群体性事件。十九大信访维稳安保工作实现“三零六无”工作目标。广安连续8年被评为“全国网民留言办理工作先进单位”，“12345”政府服务热线群众来电办结率达到99%。健全立体化社会治安防控体系，新增农村视频监控点位1701个，天网高清监控点位达到4004个。大力推进“明厨亮灶”工程，启动食品药品“智慧”监管。强化安全生产责任，狠抓重点行业领域安全生产监管，未发生重大及以上安全生产事故。国防动员和后备力量建设、双拥共建工作得到加强，征兵“五率”考评名列全国前列、全省第一。工会、青少年、妇女儿童、关心下一代、老龄、残疾人、红十字、慈善等事业发展得到加强，人民防空、住房公积金管理、贸促、外事、侨务、港澳台事务、民族宗教、社科、档案、地方志等工作取得新成绩，民主党派、工商联等作出新贡献。

同时，我们也清醒认识到，广安经济社会发展中还存在一些困难和问题：产业规模、产业层次与高质量发展阶段不相适应，工业化进程远未完成，确立和奠定广安在成渝经济区中地位的产业支撑还不强；优质普惠的教育、医疗、养老等公共服务供给不足，社会事业发展还有不少短板要补；政府债务、金融、安全生产等领域的风险不容忽视；转变政府职能、改进政府工作方式、提高工作效率，还有大量工作要做。我们一定高度重视，有计划、实打实逐步解决。

各位代表，经过设地建市25年的夯基蓄势，特别是经过近几年的快速增长，广安发展正处于厚积薄发、高点跃进的重要关口，优势更加凸显、后劲更加充足、前景更加广阔，我们有信心、有条件更加奋发有为地去谋求和实现新时代广安更大发展！

把新时代广安发展宏伟蓝图变为美好现实

中国特色社会主义进入新时代，我国经济发展由高速增长阶段转向高质量发展阶段。市委五届四次、五次全会以习近平新时代中国特色社会主义思想为指导，贯彻落实党的十九大精神，按照省委十一届二次全会决策部署，绘就了到本世纪中叶广安发展宏伟蓝图。从现在到2020年的全面建成小康社会决胜期，打好防范化解重大风险、精准脱贫、污染防治“三大攻坚战”，加快建设发展水平高、产业体系优、创新能力强、市场机制活、协调发展好的经济强市，为广安实现社会主义现代化打下决定性基础。从2020年到2035年，在全面建成小康社会的基础上，再奋斗十五年，加快建设美丽繁荣和谐广安，同步基本实现社会主义现代化。从2035年到本世纪中叶，在基本实现现代化的基础上，再奋斗十五年，全面建成美丽繁荣和谐广安，为我国建成富强民主文明和谐美丽的社会主义现代化强国贡献力量。

一届政府任期五年，应该、也完全能够集中力量干成几件大事。我们要按照市委五届四次、五次全会系列重大决策部署，认真落实市委“一个愿景、两个跨越、三大发展战略和军民融合发展战略”总体谋划，准确把握高质量发展阶段新特征，敏锐捕捉发展新机遇，坚持把准大势和顺应大势抓发展，坚持创造优势和突出优势抓发展，坚持着眼长远和奠基长远抓发展，主动有为推进质量变革、效率变革、动力变革，紧紧抓住几件事关全局的大事集中用力、深耕细作，一步一个脚印地向市委确立的新时代广安发展宏伟目标迈进。

谋划和发展几个决定广安在成渝经济区地位的支撑产业。从创造产业生态入手，把规划建设产业功能区作为主路径主抓手，全力抓几个看得清、能干成、有前景的重大产业，以此来博得广安在成渝经济区国家重要增长极中的应有地位。近期，选几个适合在广安发展的军民融合主打产品先突破，逐步把广安建成全省乃至西部地区重要的智慧高端制造基地；借力天府新区在成都规划建设广安生物医药产业研发基地，在广安与成都天府国际生物医药城共建生产园区，逐步把广安建成国家级生物医药高新技术产业开发区。同时，加强对现有支柱产业的培育壮大和提档升级。

做响小平故里和华蓥山这两张广安最具吸引力的旅游品牌。以对小平同志无比敬仰和对历史高度负责的态度，精心保护小平故里自然生态和历史文脉，保持一份特有的宁静和庄严。依托小平故居连线规划建设协兴老街（北山小学堂）、翰林院子、道台院子以及佛手山3公里长的精品旅游景区，用小平同志真真实实成长足迹吸引人、留住人来教育人，尽快建成世人缅怀世纪伟人、追寻中国改革开放和现代化建设历程的重要目的地。充分利用华蓥山海拔1000米以上形成的温差优势和毗邻重庆的区位优势，秉承“财富传世”理念雕琢一砖一瓦、一草一木，在川渝地区“大火炉”周边规划建成一座“大凉城”，确保把华蓥山打造成为川渝地区消夏避暑休闲度假第一高端旅游品牌。

建设一个广安人民更加引以为豪的城市。按照“创新、生态、智慧、人文”城市新理念，高水平规划建设一个城市核心区，完善功能配套，塑造形象地标，让我们的城市品位高起来、产业兴起来、人气旺起来、生活乐起来，在不久的将来成为区域性人才和创新要素聚集地、高品质生活首选地、休闲消费重要目的地。加快推进广安到成都动车公交化运行，积极推进与重庆主城区快速交通连接，创造和提升区位优势；加强与周边城市特别是重庆产业融合发展，创造条件承接产业转移，扩大旅游、农产品、商贸等市场合作，让我们广安以蓬勃发展之势崛起于成渝城市群。

绘就一幅人们向往的广安田园生活画卷。在广安现代农业发展良好基础上，精心编制和实施乡村振兴战略规划，逐步使未来的村落在科学规划引导下成为点缀山水田园的高品质生活区，未来的农业通过走规模化集约化之路成为有奔头的产业，未来的农民成为懂技术、善经营的体面职业，未来的农村在传统农耕文明和现代工业文明交融中成为人们寄情山水、养老养生、创新创业的美丽家园。

为广安人民办区域性最好的教育、办区域性最好的医疗、办区域性最好的养老。实施“全域公办学前教育示范市三年行动计划”，到2020年新增公办幼儿园160所、新增公办幼儿园学位数61950个，全市80%以上适龄儿童能到公办幼儿园上学；新（改、扩）建义务教育阶段学校59所，基本消除“大班额”；显著提升现有的4所省一级示范高中办学质量，支持每个区市县至少办1所优质高中，让孩子在广安就能接受良好教育。引进优秀人才，配备先进设施，打造温馨环境，做强3所市级医院，做优6所县级医院，做实一批基层医疗卫生机构，让人们在广安就能享受优质医疗。加快构建居家为基础、社区为依托、机构为补充、医养相结合的养老服务体系，探索用国有办法和新模式办高水平的养老服务企业，示范带动多层次、多元化养老服务市场发展，让广安老人生活安乐、颐养天年。

各位代表，万丈高楼层层起，我们各级政府要在市委的坚强领导下，以强烈的使命感责任感紧迫感，专注和干好“应该干、能够干”的大事，不断成就和奠基广安一项一项事业，书写“广土安辑”时代新篇！

夺取广安贯彻党的十九大精神开局之年重大胜利

2018年是贯彻党的十九大精神开局之年，是改革开放40周年，是实施“十三五”规划承上启下的关键一年。今年政府工作的指导思想：以习近平新时代中国特色社会主义思想为指导，深入贯彻党的十九大和中央经济工作会议精神，全面落实省第十一次党代会、省委十一届二次全会、省委经济工作暨全省金融工作会议重大部署，保持专注发展转型发展战略定力，坚持稳中求进工作总基调，坚持新发展理念，紧扣社会主要矛盾变化，按照高质量发展的要求，认真落实市委“一个愿景、两个跨越、三大发展战略和军民融合发展战略”总体谋划，抓好庆祝改革开放40周年机遇，坚定推进供给侧结构性改革，统筹推进稳增长、促改革、调结构、惠民生、防风险各项工作，大力推进改革开放，打好防范化解重大风险、精准脱贫、污染防治“三大攻坚战”，促进经济社会持续健康发展，为中国梦广安篇章增光添彩。

全市经济社会发展主要预期目标：地区生产总值增长8%，地方一般公共预算收入增长10%，规模以上工业增加值增长9%，全社会固定资产投资增长10%，社会消费品零售总额增长10%，城镇居民人均可支配收入增长8%，农村居民人均可支配收入增长9%，单位GDP能耗降低3.5%，空气质量优良率78.6%以上，国控断面和城市集中式饮用水水源地水质达标率100%。

扩大有效投资。扎实推进以产业为主的“项目年”工作，发挥投资对优化供给结构、稳定经济增长的关键作用。实施省市县重点项目700个以上，力争完成投资1000亿元。推动省道407华蓥段改建等216个省市重点项目开工建设，加快省道208邻水段、西部硅谷等298个省市重点项目建设，促进岳池创新药物公共服务中心等196个省市重点项目竣工投产。支持企业扩大再生产、技术改造与创新、新产品开发，提高技改投资在投资中的比例。落实和完善促进民间投资政策，促进民间投资持续稳定增长。加大金融支持实体经济力度，扩大直接融资规模。

推进先进制造业强市建设。加快发展新材料、新一代信息技术、新能源及新能源汽车等战略性新兴产业，推动聚酰亚胺、聚苯硫醚有机高分子材料等产业化应用，以及杂卤石型钾盐开发利用，建成广安北斗思源智慧城市体验馆以及北斗时频、北斗+NB模组生产线，推进甲醇制芳烃等新能源项目建设，促进比亚迪新能源汽车项目布局广安。

发展壮大电子信息、生物医药、高端装备制造、精细化工等支柱产业，推动手机整机、平板电脑等电子终端产品提档升级，打造智能终端产业集聚区；建设成都·广安生物医药研发基地，规划建设广安（岳池）医药产业园15平方公里拓展园；规划和发展适合广安的高端装备制造业，依托比亚迪云轨项目建设轨道交通产业基地，依托汽摩配套发展整车制造产业，规划建设通用航空产业基地；推进草铵膦、亚磷酸等项目建设，引导广安经开区化工产业健康发展。

提档升级能源电力、建材、食品饮料等传统产业，积极推进燃煤机组生物质耦合发电试点，坚持以国有方式规范化规模化开发嘉陵江、渠江砂石资源和华蓥山非煤矿山资源，科学规划和发展旅游食品、保健食品等“广字号”特色食品饮料产业集群。

实施乡村振兴战略。坚持农业农村优先发展，按照“产业兴旺、生态宜居、乡风文明、治理有效、生活富裕”总要求，贯彻城乡融合发展理念，以区市县为单位编制乡村振兴战略规划。落实第二轮土地承包到期后再延长三十年政策，按照“三权分置”要求深化农村土地改革。

深化农业供给侧结构性改革，突出抓好建基地、搞加工、创品牌等重点任务。扩升“111”工程，支持区市县集中连片规划建设乡村振兴示范基地。在部分区市县率先探索，整合涉农资金，规划建设一批规模养殖与规模种植循环发展、统筹推进的现代种养业基地。重视支持市属涉农国有企业参与现代农业发展，组建一批体现政府主导和支持的混合所有制企业，探索直接带动农民致富的经营模式，既让小农户融入大市场，更让小农户长久增收致富。从塑造广安农产品的质监品牌入手，面向重庆等市场抓一批主打农产品，严把使用“华蓥山”公用品牌产品质量关，稳步扩大高端市场份额。深化供销合作社综合改革，把供销社作用做实。全面落实粮食安全行政首长责任制。

坚持产村相融、农旅融合，推动乡村各类资源景观化，深入实施休闲农业提升工程和千村万景乡村旅游富民计划，打造一批农业主题公园、休闲农庄和A级景区。实施“四好农村路”创建，新（改）建县乡道150公里、村级公路370公里，建设旅游路、产业路80公里。推进农村土地开发整理、城乡建设用地增减挂钩、工矿废弃地复垦等项目，腾挪和用好建设用地指标。

健全自治、法治、德治相结合乡村治理体系，培养造就一支懂农业、爱农村、爱农民“三农”工作队伍，提高发展农业、服务农民、稳定农村的能力。

发展现代服务业。着眼增加有效供给，抓好现代物流、电子商务、科技服务、健康养老、通信服务等新兴先导型服务业，发展现代金融、现代旅游、文化创意等新业态，提高服务业在三次产业中比重。

提前研究与重庆主城区快速交通连接、时速350公里高铁通过广安等带来的重大影响，慎重谋划、精准定位、巧妙构建广安服务业发展新格局，促进与周边地区合作共赢发展。围绕“买得到”，支持建设档次高、品种全的专业市场和骨干卖场，丰富中高端商品供给，让市民消费留在广安。围绕“卖得出”，重视线上线下融合，培育和引进一批电商物流企业，推出一批地方特色商品品牌，扩大广安特色优质商品市场占有率。围绕“愿意来”，有针对性开发具有地方特色的商品和服务，重视发展高端休闲旅游产业，办好一批会展、美食、特色文化等节会，吸引周边地区到广安消费。围绕“放心买”，创新和落实扩大消费的政策，整顿和规范市场秩序，创建放心舒心消费城市。

抓住庆祝改革开放40周年机遇，启动省级红色旅游融合发展示范区建设。高水平实施小平故里周边环境改造及协兴镇景点景区整合提升工程。按照“红色、生态、健康、美丽、避暑胜地”定位，推进华蓥山旅游整体开发，实施绿化、交通、水源工程、土地利用等专项规划，启动“704-椿木坪”片区基础建设。办好2018年华蓥山旅游文化节。推动武胜白坪-飞龙乡村旅游度假区等优化提升，加快岳池农家生态文化旅游区建设。

深化改革扩大开放。扎实抓好“三去一降一补”年度任务，引导煤矿企业“关停并转”，有效化解煤炭过剩产能；消化商品住房库存60万平方米以上；鼓励引导企业直接融资，降低杠杆率；落实“营改增”试点等减税降费政策，推进水电气等资源性产品价格改革，降低实体经济成本。实施全面创新改革“一号工程”，结合广安实际落实省委“9张清单”，推进国家知识产权试点城市建设工作。继续深化“放管服”改革，动态调整完善权责清单，全面推行行政许可标准化建设，推动相对集中行政许可权改革，推进“减证便民”专项行动，做好“互联网+政务服务”。深化投融资体制改革，完善投资项目审批核准备案制度，规范并完善政府和社会资本合作、特许经营管理。规范和做大小平故里“1+N”基金群。深化国有企业“三项改革”，构建激励与约束相统一机制，把企业自主经营权落实到位，把国有资产监管到位。

认真落实市委庆祝改革开放40周年活动工作方案，推动开放合作再上新台阶。坚持把招商引资作为推动经济发展的生命线，改进招商引资方式，更加注重营造产业生态，把准产业发展规律，善于用基金引进产业项目。创新开放合作模式，深化与重庆、成都、深圳、天津、北京中关村等区域合作，在对接和融入大都市经济圈中借势借力发展。支持邻水县打造川渝合作“桥头堡”，在深化与重庆合作上创新政策、率先突破、走出新路。加强贸易投资促进，运营好外贸保税仓、外贸综合服务“双平台”，实现外贸进出口总额20亿元以上，实际利用外资4500万美元以上。

完善城乡基础设施。注重城乡要素互动、功能互补、联动互进，促进城乡均衡协调发展。善于经营城市，重视用高水平规划来促进土地等城市资源增值，重视从产业发展需要来扩建城市新区，重视完善公共服务设施来提升城市品位。针对庆祝改革开放40周年，集中抓几个主城区环境提升项目。

促进组团城市互动发展，研究提出主城区提升品质和增强承载能力实施方案。实施交通强市战略，推进广（安）忠（县）黔（江）铁路前期工作，启动镇广渝高速公路通江至广安段和长（寿）邻（水）渝（北）快速通道建设，加快过境高速公路东环线及渝广高速支线建设，推进广安主城区与前锋区、华蓥市、岳池县等组团城市同城化发展，加快广邻、广武快速通道项目进程，确保广华快速通道、港前大道建成通车，完成广安至华蓥输水管线建设。深化拓展“百镇建设行动”，完成50个特色集镇基础设施投资14.6亿元，力争培育申报国家级和省级特色小镇各1个、“百镇建设行动”扩面增量5个。

加强城乡生态环境设施建设。做好国家卫生城市迎接复审和全国文明城市保持常态工作。市主城区建成公共停车泊位3000个。开展城市生态修复、城市修补，加快A号桥、迎宾大道绿化景观提升工程、莲花大道地下综合管廊、广前大道城区段改造等项目建设。有计划推进老旧小区供水、供电、供气、雨污分流设施改造并限期完成。建成广安市生活垃圾焚烧发电厂二期及广安市污泥处置中心。抓好场镇设施配套，建成污水主管网103公里。整治提升场镇环境，改变场镇无序建设、规划和管理不到位的状况。推进5座中型水库建设，完成猫儿沟水库大坝填筑工程，回龙寺和应家沟水库大坝填筑工程完成50%以上，启动向阳桥和龙滩水库大坝填筑工程。

推进生态优先绿色发展。牢固树立和切实践行绿水青山就是金山银山的理念，彻底转变发展观念，更加重视在发展中把生态建设好、环境保护好。深入推进嘉陵江流域国家生态文明先行示范区建设，从源头上把好生态环保关，坚决防止在发展过程中新增生态环境隐患。新实施发展项目充分考虑环保成本和项目落地关联影响，注意为持续发展留空间。

打好污染防治攻坚战。完成中央和省级环保督察问题整改。开展工业污染减排、抑制城市扬尘、压减煤炭消费总量、治理机动车尾气污染、控制秸秆焚烧“五大工程”，PM2.5、PM10年平均浓度分别控制在75微克/立方米、42.4微克/立方米以内。全面落实河长制和最严格水资源管理制度，加强污染水体整治、良好水体保护、水污染防治设施建设、饮用水环境安全保障，确保国控省控断面水质稳定达标、重点小流域和湖库水质持续改善。深化全域“洁净水”行动，加快实现县城污水“零直排”，把人口规模较大的集镇纳入消除直排范围管理并限期达标。加强固体废物、危险废物、医疗废物监管，以及化肥、农药使用指导，保障土壤环境稳定向好。

深入实施新能源汽车“免费充、绿色行”行动，鼓励发展新能源汽车、共享汽车等低碳产业。逐步建立市场化、法治化、常态化的落后产能退出机制，开展重点企业节能节水行动和能效领跑者引领计划。统筹谋划、扎实推进华蓥山区山水林田湖草生态保护修复试点工程。

持续推进造林绿化，以重要景区、交通干线、江河湖库以及城市、场镇为重点，划定区域、选好树种，加快城乡绿化美化。实施华蓥山“三年会战、漫山红遍”行动计划，在“704-椿木坪”、西天乡经天意谷、石林至天池等区域造林3.5万亩。

聚力全域贫困县摘帽。坚持把脱贫攻坚作为头等大事来抓，念兹在兹、唯此为大。下足“绣花”功夫，深化精准扶贫、精准脱贫，完成岳池、武胜、邻水3个贫困县摘帽和215个贫困村退出、4.4万名贫困人口脱贫任务，在全省率先实现全域贫困县摘帽。

强化精准施策，聚焦“两不愁、三保障”，有针对性地做好发展生产、就业帮扶、易地搬迁、低保兜底、医疗救助等工作。拓展就业渠道，增加就业岗位，确保有劳动能力的贫困家庭至少有1人就业。启动实施“农村土坯房改造行动”，推动13500户农村土坯房整治、4500户农村危房改造。注重教育扶贫，确保所有适龄儿童、少年能就近入学。加强医疗卫生扶贫，落实“十免四补助”等健康扶贫政策，贫困人口县域内住院治疗和慢性病门诊维持治疗个人支付占比不超过10%。巩固农村低保线和国家贫困线“两线合一”成果，探索建立支出型贫困家庭享受低保制度，构建全覆盖低保救助体系。

完善基础配套，推进贫困村饮水通村入户，新（改）建集中供水工程16处，解决40380人安全饮水问题，确保农户供水保障率达到95%以上。新（改）建扶贫公路420公里。新（改）建乡镇便民服务中心25个。完成215个计划脱贫村文化室、卫生室全面达标建设。

巩固脱贫成效，做实“回头看”“回头帮”工作。对411个已退出村和3个摘帽区市，同步巩固脱贫成果，确保脱贫摘帽对象持续稳定脱贫。对25.3万名已脱贫群众，有针对性地落实巩固措施，确保脱贫不脱帮扶、不脱政策、不脱项目。

做实民生事业。花在民生上的钱每一分都是值得的，不能政府省小钱让老百姓花大钱。把贯彻落实以人民为中心的发展思想转化为我们各级政府主动解决民生难题的实际规划、实际投入、实际行动，让广安老百姓得到更多实惠、看到更大变化。

坚持办好民生实事，确保完成省定十项民生工程和20件民生实事，以及助力“三农”、扶贫解困、环境保护等八大类市自办民生工程。全年力争新增城镇就业3.6万人，城镇登记失业率控制在4%以内。全面实施全民参保计划，推进城乡居民养老保险基金集中管理，实施建档立卡未标注脱贫的贫困人口、低保对象、特困人员参加城乡居民基本养老保险个人缴费财政代缴政策。启动建设居民家庭经济状况核对信息平台，探索推进城乡低保标准并轨试点。完成城镇危旧房棚户区改造开工11189套、基本建成7045套、竣工6759套。建立与社会救助体系相衔接的慈善事业服务网络。积极推进“厕所革命”，制定厕所建设管理以奖代补管理办法，新（改）建城乡公厕200座。支持工会、共青团、妇联、残联等群团组织结合职能谋划和做好惠民工作。

办区域性最好的教育。今年，以市主城区和各县城老城区、拆迁安置集中居住区、人口多乡镇为优先，启动建设公办幼儿园80所、建成54所，其中市主城区建成10所、各区市县城区建成26所，新增公办幼儿园学位数25710个，确保全市公办幼儿园学位数占比由26.4%提高到50%以上。启动实施“化解大班额三年攻坚计划”，新（改、扩）建义务教育阶段学校22所，其中市主城区3所。启动广安中学创“中华名校”试点，高水平推进广安友谊中学、邻水中学等省一级示范高中建设，推动各区市县集中力量至少建1所优质高中。落实中高考改革政策，推进普职融通改革，促进城乡义务教育一体化发展。支持广安职业技术学院创办应用型本科院校。重视培养和引进高水平师资力量，加强师德师风建设。扎实开展健康跑、书法、足球“三进校园”活动，认真筹办好市第四届中小学艺术节、第十五届中国中学生排球联赛、广安国际红色马拉松赛。申办省第十四届运动会。

办区域性最好的医疗。按照“做强龙头医院、做优县级医院、做实基层医疗”的思路，优化医疗资源配置，深化医药卫生体制改革，全面提升医疗技术水平和服务能力。实施“医疗卫生人才512计划”，加大医疗卫生专业技术人员、高职称高学历人才和急需紧缺专业人才引进力度。支持市人民医院建成省级医学重点专科，确保市人民医院儿科及第二住院大楼投入使用。振兴发展中医药事业，推动成都中医药大学与市中医院深化合作，加强专业人才引进和重点专科建设。高标准推进市妇女儿童医院建设，做好妇产儿科医生引进和培养工作。启动公立医院薪酬制度改革，巩固完善分级诊疗制度。健全覆盖城乡的疾病防控和预防保健体系。

办区域性最好的养老。扩大养老服务有效供给，开工建设广安市养老服务中心、前锋区综合社会福利救助中心等融合养老、康复、医疗的公办养老机构。推动华蓥市农村养老服务体系建设试点，加快农村敬老院、日间照料中心公建民营建设。鼓励引导社会力量参与发展养老服务业，推广“嵌入社区式”养老驿站等居家养老服务模式，引进知名品牌养老企业落户广安，开展居家社区养老社会化信息化服务。

繁荣发展文化事业。继续做好“优秀剧目进广安”、城市音乐会、“网络春晚”等文化惠民活动，抓好反映广安历史文化、风土人情剧目创排工作。分门别类编制好广安文物保护专项规划，实施广安白塔、文庙等省级重点文保单位维修。完成市博物馆建设。抓好重点档案保护抢救和档案数字化建设。推进“高清四川·智慧广电”示范市建设，实施数字化终端、宽带乡村等建设项目。做好地方志工作。

加强和创新社会治理。从源头上预防和减少矛盾发生，完善不稳定因素排查和矛盾纠纷多元化解机制，推进民商事调解、仲裁工作，做到重大决策社会稳定风险评估全覆盖。健全警务实战指挥体系，推进派出所改革，提升公安核心战斗力，建设现代警务体系。扎实开展扫黑除恶专项斗争，大力实施“2018平安广安”行动，严厉打击黄赌毒、黑拐枪、盗抢骗和经济领域等违法犯罪，开展净网行动。加强公共安全视频监控联网应用建设，实施“雪亮工程”项目村400个，健全立体化信息化社会治安防控体系。深化“法律七进”，做好“七五”普法中期督导迎检工作。

健全公共安全体系。完善应急管理基础数据库，增强防灾减灾救灾能力。实施重大地质灾害治理和中小型应急排危治理，抓好地质灾害避险搬迁安置。推进安全生产领域改革发展，理顺监管体制，完善责任体系，突出重点行业监管执法，减少一般事故，遏制较大事故，杜绝重特大事故。建立覆盖全市的一体化食品药品智慧监管平台，保障群众“舌尖上的安全”。探索构建米、面、油等大宗食品面向全国知名企业集中直购制度，让全市大中小学生食品安全真正有保障。

加强新时代军政军民团结，扎实做好国防动员和民兵预备役工作，深化民兵调整改革，积极争创“全国双拥模范城”五连冠。

立足推进新时代广安发展加强政府自身建设

全面加强政府系统党的建设。坚定以习近平新时代中国特色社会主义思想统领各方面工作，持续深入学习贯彻党的十九大精神，认真开展“不忘初心、牢记使命”主题教育，树牢“四个意识”，坚定“四个自信”，坚决维护以习近平同志为核心的党中央权威和集中统一领导。政府工作中严格执行党的纪律规定，对中央、省委大政方针不搞选择性执行，抵制和克服分散主义、自由主义、本位主义、山头主义、地方保护主义，不搞上有政策、下有对策。坚持在市委领导下开展政府工作，坚决落实市委决定，定期汇报经济社会发展情况，及时、主动报告工作中的重要举措并提出建议。全面加强政府系统党风廉政建设，切实履行主体责任，注重从源头上预防腐败发生，建设廉洁政府。

建立政府抓落实和执行工作体系。建立规范化制度化政府工作流程，让政府系统干事有“规范”、评价有“标准”、运行有“效率”、服务有“温度”。重视发挥政府班子整体功能，坚持行政首长负责制与民主集中制有机结合，尊重和支持分管领导履职干事。推行局长（主任）工作规范，把政府部门主要负责人基本职责和工作要求梳理出来、确定下来、执行到位。倡导“八项工作法”，即工业流程工作法、事前想透工作法、问题入手工作法、先找榜样工作法、项目包干工作法、清单管理工作法、守住底线工作法、算账决策工作法，系统解决政府工作中存在的突出问题。对照“四风”问题十种新表现持续改进政府作风，大兴调查研究之风，反空想、反空转、反空谈、反空泛，力戒形式主义官僚主义。

提高政府驾驭和防控风险的能力。善待和用好政府债务，把防范化解地方债务隐性债务风险列入政府重点督查事项，按照中省要求摸清负债总量和结构，严防违规或变相违规举债，遏制隐性债务增量。建立对各区市县（园区）新增债务的动态监控机制。建立健全地方金融监管机构，加强金融风险防控。开展涉金融债权案件专项执行年活动，整治非法集资等金融乱象，坚决制止违法违规融资担保行为。常态化关注企业债务、自然灾害、生态环保、安全稳定等重点领域风险，制定完善应急预案，防止风险失控。

自觉接受各方监督。强化行政权力制约监督，深入推进依法行政，将政府工作全面纳入法治轨道。依法接受人大及其常委会法律监督、工作监督，自觉接受政协民主监督，认真办理人大代表建议和政协委员提案。主动接受社会和舆论监督，支持监察和司法监督，重视审计监督。深化政务公开和政府信息公开，推行政务信息公开清单管理，利用现代信息手段提升服务水平，认真处理群众来信来电来访并改进工作。认真听取民主党派、工商联、无党派人士和各人民团体的意见建议，群策群力、协调一致把广安建设好。

各位代表！

肩负新时代广安更大发展重任，我们一定紧密团结在以习近平同志为核心的党中央周围，以习近平新时代中国特色社会主义思想为指导，在省委、省政府和市委的坚强领导下，不忘初心、牢记使命，开动脑筋、务实苦干，为加快建设美丽繁荣和谐广安、实现中华民族伟大复兴中国梦而努力奋斗！
